# Benign Cementoblastoma Associated with an Impacted Third Molar inside Maxillary Sinus

**DOI:** 10.1155/2018/7148479

**Published:** 2018-11-19

**Authors:** Rafael Correia Cavalcante, Maria Fernanda Pivetta Petinati, Edimar Rafael de Oliveira, Isabela Polesi Bergamaschi, Nelson Luis Barbosa Rebelatto, Leandro Klüppel, Rafaela Scariot, Delson João da Costa

**Affiliations:** ^1^Oral and Maxillofacial Surgery Resident at Federal University of Parana, Curitiba, Brazil; ^2^Dental Clinic Mastering Degree Student at Federal University of Paraná, Curitiba, Brazil; ^3^Professor of Oral and Maxillo-Facial Surgery Department at Federal University of Paraná, Curitiba, Brazil; ^4^Professor of Oral and Maxillo-Facial Surgery Department at Positivo University, Curitiba, Brazil

## Abstract

**Introduction:**

Cementoblastoma is a rare and benign odontogenic mesenchymal tumor, often characterized by the formation of cementum-like tissue produced by neoplastic cementoblasts attached to or around the roots of a tooth.

**Case Report:**

22-year-old male patient was referred to the Federal University of Paraná after occasional finding on a routine panoramic radiograph. Clinical examination suggested no alterations. Medical and family history presented no alterations as well. Computed tomographic (CT) showed the presence of a radiopaque area associated with the roots of the impacted third molar measuring 15 mm × 10 mm inside the left maxillary sinus. The treatment plan suggested was to surgically remove it under general anesthesia. An intraoral approach was conducted, using the Newmann incision from the superior left first molar to the retromolar area with anterior and posterior relaxant incisions. Using a Caldwell-Luc access next to the maxillary tuberosity region, the maxillary sinus was exposed and the calcified mass attached to the roots of the tooth was reached. Pathological mass removed was sent for histopathological investigation. Examination revealed dense, mineralized, cementum-like material and vascular soft tissue areas that consisted of cementoblasts. One-year follow-up shows no recurrence and absence of symptoms.

## 1. Introduction

Benign cementoblastoma (BC) is a rare and benign odontogenic mesenchymal tumor, often characterized by the formation of cementum-like tissue produced by neoplastic cementoblasts attached to or around the roots of a tooth. It is considered to be the only true neoplasm of cemental origin [1, 2]. It represents a very small proportion of all odontogenic tumors, with a percentage of less than 1%. The World Health Organization first named this neoplasm “benign cementoblastoma” and also “true cementoma” in their 1971 classification. This terminology was altered in 2005, and the benign prefix was dropped because there is no malignant neoplasm originating from cementum tissue [2].

These tumors primarily affect young adults in the second and third life decades, with approximately 50% occurring under 20 years old and approximately 75% occurring under 30 years old [3]. Although males are affected slightly more, there is no significant sex predilection. The neoplasm exhibits a slow but limitless growth pattern, and the mandible is involved more often than the maxilla. Typically, the lesion is seen on the posterior region of the mandible and commonly involves the mandibular first molar [3, 4]. Its typical appearance on panoramic radiographs is a large radiopaque mass in continuity with the roots from the teeth which it arose [4]. BC is encapsulated and this translates radiographically as a thin, uniform lucency around the periphery of the tumor. The density of the cemental mass usually obliterates the radiopaque details of the roots. The radiographical appearance is characteristic and usually pathognomonic [4, 5].

Cementoblastoma histopathological presentation is similar to the osteoblastoma one; however, the main distinction feature is the fusion of the tumor with the teeth involved. The major part of the lesion is formed by a mineralized mass with gaps irregularly positioned as well as prominent basophilic reverted lines [3].

Frequently, multinucleated giant cells and blastic cells are within the margins of the mineralized mass. In rare cases, cementoblastomas could infiltrate within the pulp and radicular canals of the teeth involved or associate with maxillary wisdom teeth [3, 6].

Treatment of lesion is well defined as being total excision of teeth together with calcified mass. Surgical excision of the calcified mass with root amputation followed by endodontic treatment of the teeth involved may also be considered as a treatment modality [7]. Its total relapse rate is reported to be 22%, and the removal amount is directly related to the lesion relapse. Total excision of the teeth, as well as the mineralized mass, minimizes but does not exclude the relapse possibility [8].

The aim of the present paper is to report a rare case of BC within maxillary sinus associated with an impacted third molar and its treatment.

## 2. Case Report

**Figure 1:**
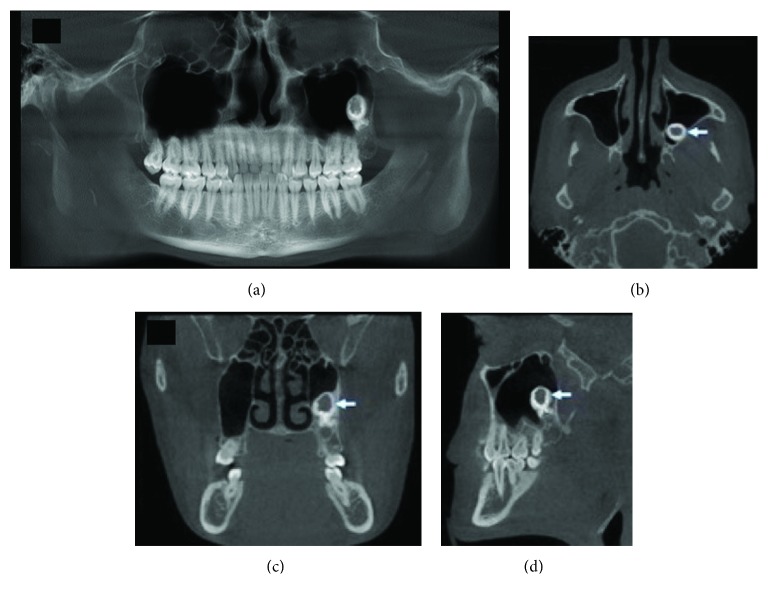
Computed tomography preoperative showing mass associated with the roots of the third molar inside the maxillary sinus. (a) Panoramic view. (b) Axial plane. (c) Coronal plane. (d) Sagittal plane.

A 22-year-old male patient was referred to the Oral and Maxillo-Facial Surgery Service at Federal University of Paraná after occasional finding on a routine panoramic radiograph. The patient experienced no symptoms. A computed tomographic (CT) was requested and showed a well-defined hyperdense mass showing a hypodense center inside the left maxillary sinus measuring approximately 15 mm × 10 mm situated in a posterior position of this anatomical space (Figure 1). It was observed that this calcified mass was associated with the roots of the impacted third molar. Treatment proposed was the complete excision of the lesion through an intraoral approach and Caldwell-Luc access to reach the maxillary sinus.

**Figure 2:**
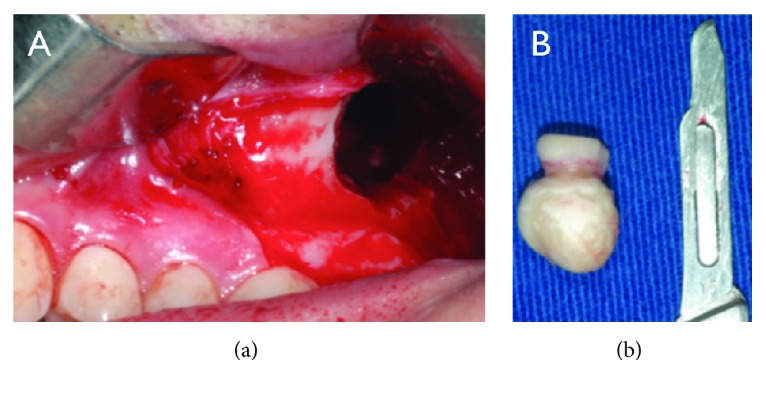
Transoperative images. (a) Caldwell-Luc access. (b) Third molar with calcified mass associated with the roots.

Under general anesthesia, an incision from the superior left first molar extending to retromolar area, with anterior and posterior relaxant incisions, was conducted to provide a sufficient access to the region of interest without causing gingival tissue tension. Osteotomy of the lateral maxillary sinus wall was conducted in order to expose its membrane. Once the sinus membrane was exposed, it was carefully detached from the bone without it disrupting until the calcified mass was reached (Figure 2). The third molar with calcified mass associated with the roots was removed.

The chosen postoperative drug therapy was cefazoline-oral (500 mg) each 8 hours during 7 days, nimesulide (100 mg) each 12 hours during 5 days, and dipyrone (1 g) for each 6 hours during 3 days. Patient experienced no infection symptoms and drug therapy showed to effective in swelling and pain control.

**Figure 3:**
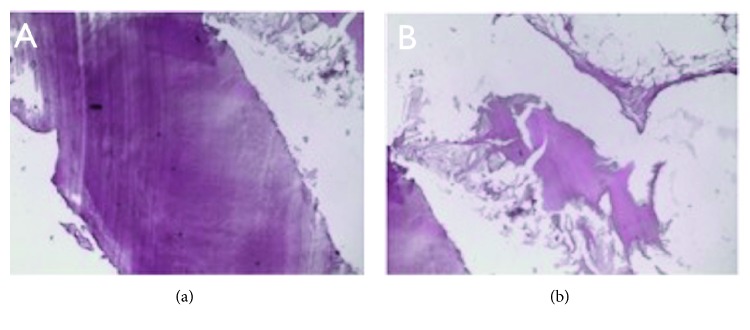
Histologic examination. (a) Cementum-like mineralized tissue (HE – 100x). (b) Numerous and voluminous cementoblastomas (HE – 400x).

Pathological mass removed was stored in 10% formalin and sent as excision biopsy for further histopathological investigation. It was fixed in 10% neutral formalin, subjected to decalcification in formic acid, bisected in a mesiodistal direction, and then processed for light microscopic examination. Histopathology showed that the calcified tumor mass was composed of sheets of cementum-like tissue with lack of interstitial tissue. The middle part of the tumor was found to be more mature and the peripheral part is more cellular. Multinucleated giant cells and blastic cells were found within the margins of the mineralized mass. The pathological mass was also associated with the yet not totally formed maxillary third molar root, corroborating the benign cementoblastoma diagnosis (Figure 3).

**Figure 4:**
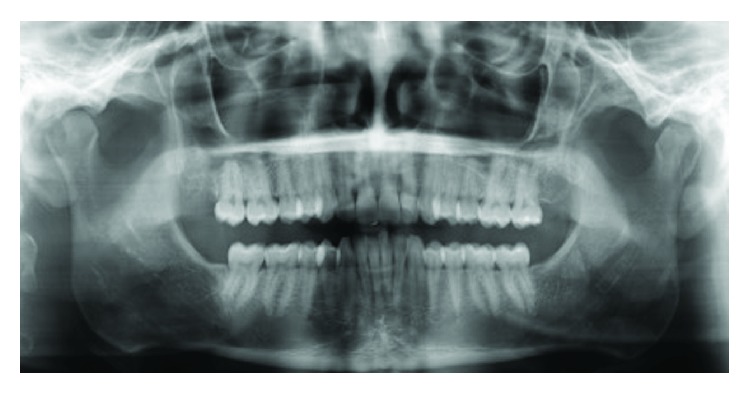
Panoramic radiography (one year postoperative).

Postoperative orthopantomogram radiography shows the success of the tumor removal (Figure 4). One-year follow-up shows no recurrence and absence of symptoms.

## 3. Discussion

First described by Dewey in 1927, BC is a benign tumor of cementoblast origin, in which cementoblasts form cementum-like disorganized tissue around the root of a tooth or rarely multiple teeth [9]. Mandible is affected in majority of cases; however, benign cementoblastomas involving multiple teeth is reported to occur more commonly in the maxilla, reflecting the high growth potential of those tumors [10].

The cementoblastoma was found in different regions of maxilla, since anterior region, associated or not with impacted teeth, to posterior maxilary region, associated with erupted premolars and molars. Its occurrence involving deciduous dentition in maxilla was found to be more associated with multiple teeth [10–12]. Different treatment types were proposed in different cases and none of them presented lesion relapse, regardless of treatment proposed. One of the reported cases was associated with an impacted left central incisor (21) in the premaxilla. Treatment proposed was total enucleation of lesion with upper central incisor extraction [13]. Depending on the lesion size, its amplitude, and its location, another proposed treatment type was lesion enucleation with teeth apicoectomy [14, 15]. Hirai et al. [14] proposed this treatment type of a benign cementoblastoma associated with an erupted canine. 18-month follow-up showed no lesion relapse and a positive prognostic on the maintained teeth [14]. Baker et al. [15] also reported enucleation of the lesion and apicoectomy but cementoblastoma was associated with an erupted maxillary right second molar. Twelve-month follow-up showed no lesion relapse and again, a positive on the maintained teeth [15].

The benign cementoblastomas are rarely associated with third molars. It was observed that the reported cases of benign cementoblastomas occurred mostly associated with mandibular third molars. In all the cases reported, pain and swelling were observed, highlighting the importance of early extraction of third molars [6, 16–18]. One of them was associated with infection and extraoral draining [16]. Treatment proposed was third molar extraction and lesion enucleation. No cases were found to be associated with an impacted upper third molar within maxillary sinus. Neelakandan et al. [19] reported a high proportion of benign cementoblastoma associated with the maxillary sinus but not inside it. Its treatment involved lesion enucleation, extraction of teeth involved as well as ostectomy of the maxillary sinus floor [19].

Panoramic radiograph and CT evaluation of the lesion reported on this case were not pathognomonic for benign cementoblastoma. It was first thought it was an unerupted third molar with an unusual morphology. Clinical evaluation showed no pain, swelling, sinusitis, or cortical expansion. General anesthesia was chosen due to the posterior and upper maxillary sinus localization of the lesion as well as its obscure etiology.

Histopathologically, the periphery of the cementum-like tissue presents more active growth, and sometimes, resembles osteoblastoma, osteoid osteoma, or atypical osteosarcoma, which are not distinctively related to tooth roots, and may be difficult to distinguish from these tumors [3, 5]. Cases of BC associated to maxillary sinus usually involve a permanent and erupted tooth with a lesion attached to its roots [19]. Our case, on the other hand, describes the lesion inside this space, posterior and high positioned related to an unerupted third maxillary molar. Between the mineralized and trabecular hard tissue, there is fibrovascular tissue with cementoblast-like cells. Tumor fusion to the tooth is the primary distinguishing feature of a cementoblastoma, as it might resemble an osteoblastoma histologically. Some authors believe that benign cementoblastoma may actually be an osteoblastoma which is attached to the root. Due to the fact that the lesion has unlimited growth potential, enucleation and extraction of teeth involved should be curative. This treatment would vary according to the lesion location, size, and teeth involved. Enucleation and apicoectomy for small to moderate BC sizes associated with erupted teeth were also found to be efficient, and tooth function was maintained [14, 15]. Literature review reported no recurrence cases.

Cementoblastomas have an unlimited growth potential and require surgical removal along with the involved tooth. The growth rate is estimated to be 0.5 cm per year. The tumor can usually be removed in 1 unit with the tooth attached to the lesion [20]. The buccal cortex around the tumor may be absent or severely thinned which may require a bone graft. Recurrence is not expected, unless a portion of the tumor is left behind [21]. Brannon and colleagues reviewed a case series of 44 recurrent cementoblastomas and recommended a peripheral ostectomy, in addition to surgical removal, to reduce the chance of recurrence. In this case, the treatment was complete excision of the third molar with calcified mass attached to the roots. Six-month follow-up shows no recurrence and absence of symptoms. An excellent prognosis is usually achieved after complete removal of the tumor [22, 23].
